# Hardware–Algorithm Co-Optimization of Weight-Update Protocols in Oxide-Based Synaptic Transistor Arrays for Neuromorphic Systems

**DOI:** 10.3390/mi17091049

**Published:** 2026-09-02

**Authors:** Yixin Cao, Jingsong Xia, Xiangyi Ding, Xin Wang, Jin Liu, Canhua Xu

**Affiliations:** 1 Shaanxi Provincial Key Laboratory of Bioelectromagnetic Detection and Intelligent Perception, Department of Biomedical Engineering, Fourth Military Medical University, Xi’an 710032, China; 2 Department of Basic Medical Sciences, Fourth Military Medical University, Xi’an 710032, China

**Keywords:** neuromorphic computing, synaptic transistor array, differential conductance-pair mapping, weight-update optimization, hardware–algorithm co-design

## Abstract

The transition from single-device characterization to array-level simulation remains a critical challenge in the development of three-terminal synaptic transistors for neuromorphic computing, as most existing simulation studies either extract parameters from a single representative device and apply them uniformly, or rely on weight-update strategies originally designed for two-terminal memristors. Here, we establish an experimentally calibrated behavioral simulation framework based on differential conductance-pair mapping (W = G^+^ − G^−^, where G^+^ and G^−^ denote the conductances of the positive and negative devices of each pair), integrating exponential long-term potentiation/depression (LTP/LTD) update rules with a posteriori screening mechanism (isValid) to systematically investigate how update polarity, step size, nonlinearity, and conductance boundaries regulate network computational efficiency. Through comprehensive simulation on the Neural Circuit Policies network, we demonstrate that the update direction must strictly align with the matrix’s role: the G^−^ channel requires unidirectional long-term depression inhibition, while the G^+^ channel can be frozen or bidirectionally updated. The optimal G^−^LTD and G^+^G^−^LTD strategies achieve accuracies of 0.9208 and 0.9481, respectively. Furthermore, we reveal a unique nonlinear gain effect under long-term depression > 0, where accuracy increases monotonically with nonlinearity level up to 0.9419. Device specification criteria are established: LTP-dominant updates favor large G_max_, while LTD-dominant updates favor high G_min_, with the G^−^LTD and G^+^G^−^LTD strategies showing accuracy fluctuations within ±0.005 across the tested boundary variations. Finally, the array-level implementation is validated through a functional-correctness check and device-parameter ablation experiments on a 23,715-weight array (47,430 differential conductance elements). This work provides an experimentally calibrated behavioral simulation platform and concrete algorithm-hardware co-design guidelines for future neuromorphic hardware, prioritizing synaptic devices with long-term depression > 0, a moderately elevated G_min_, and asymmetric resource allocation toward LTD-side optimization.

## 1. Introduction

The relentless pursuit of higher computational performance is increasingly challenged by the fundamental limitations of the traditional von Neumann architecture, where the physical separation of processing and memory units creates a notorious “memory wall” and “power wall” [1,2,3]. This bottleneck is particularly acute for data-intensive tasks such as artificial intelligence and machine learning, which demand massive parallelism and frequent data shuffling [4,5]. In response, neuromorphic computing, which mimics the brain’s highly parallel and energy-efficient information processing, has emerged as a transformative paradigm [6,7]. At the heart of this paradigm lie in-memory computing devices, which integrate storage and computation within a single unit, thereby bypassing the need for constant data transfer between separate components [8,9,10].

Although three-terminal synaptic transistors share the nonlinear, saturation-limited LTP/LTD conductance dynamics with their two-terminal counterparts—indeed, the LTP/LTD operation of the GMX device in Ref. is driven by hybrid light/electrical stimulation in a manner similar to optoelectronic two-terminal synapses—the independent gate terminal still provides distinct architectural advantages for array integration [11,12,13], including crosstalk-free cell selection and an additional degree of freedom for conductance modulation [14]. The differential mapping and update-protocol design presented in this work are formulated at the level of the parametric LTP/LTD update equations and are therefore applicable to both device classes. In our previous work, we proposed and demonstrated a high-performance optoelectronic synaptic transistor based on GeO_x_-coated MXene (GMX) nanosheets [15]. The device leverages a heterostructure of GeO_x_ and zinc tin oxide (ZTO), enriched with oxygen vacancies, to achieve non-volatile photocurrent generation under visible light. Under visible-light pulses, photogenerated electrons are captured by the GeO_x_/ZTO interface states and ionized oxygen vacancies, producing a nonvolatile photocurrent that gradually increases the channel conductance and realizes LTP; under gate-voltage pulses, the trapped carriers are released/de-trapped and the accumulated conductance is partially erased, realizing LTD [16,17]. This optoelectronic trapping/de-trapping mechanism is consistent with the working principles of optoelectronic synaptic devices widely reported in the literature, and it naturally explains the exponential saturation characteristics of the measured update curves. With its tunable long-term potentiation/depression (LTP/LTD) characteristics and excellent uniformity, the GMX-based synaptic transistor has proven to be a promising candidate for constructing efficient neuromorphic systems [15,18].

Despite these advances at the single-device level, a critical gap remains in the transition from device characterization to array-level simulations [19,20]. Most existing simulation studies either extract parameters from a single representative device and apply them uniformly across the entire neural network, or rely on weight-update strategies originally developed for ideal software-based networks or two-terminal memristors [21,22]. These approaches, however, fail to capture the inherent device-to-device variability and the specific constraints imposed by the three-terminal architecture when deployed in a crossbar array. More importantly, the unique role of the gate terminal in a three-terminal synaptic transistor—which can serve as a natural switch or modulator—has not been fully exploited in the design of weight-update mechanisms [23,24]. Consequently, there is an urgent need for a dedicated simulation framework that not only models array-level hardware realities but also optimizes the weight-update protocol to fully leverage the structural advantages of three-terminal devices.

Here, we establish an experimentally calibrated behavioral simulation framework for three-terminal synaptic transistor arrays, based on the experimentally extracted LTP/LTD parameters from our previously reported GMX devices [15]. The framework uses compact parametric update equations rather than full device-physics solvers, following the standard practice of compact behavioral modeling in circuit-level co-design; all parameters are fitted from measured device data. Our approach maps each synaptic weight to the difference in conductance of a pair of devices: W = G^+^ − G^−^, where G^+^ and G^−^ denote the conductances of the positive and negative devices of the pair, respectively, and W is the resulting effective synaptic weight. Each device conductance is physically bounded within [G_min_, G_max_], where G_min_ and G_max_ are the minimum and maximum programmable conductances of the synaptic device, respectively. This differential pair configuration mimics the differential pairing commonly used in hardware arrays. We systematically investigate how key device parameters—specifically, the update polarity constraints, step size, nonlinearity (NL), and conductance boundaries (G_max_/G_min_)—affect the performance of neuromorphic computing tasks. To ensure reliable and efficient updates, we introduce an “isValid” screening mechanism to filter out ineffective updates that deviate from the intended weight change, and we further optimize the update protocol by incorporating polarity-specific rules, step-size scaling, and an analysis of nonlinearity effects. The efficacy of our proposed framework and optimization strategies is validated using the ICRA2020 LiDAR obstacle-avoidance dataset, coupled with a Neural Circuit Policies (NCP) network [25], which is well-suited for robust decision-making in autonomous driving scenarios. Our results demonstrate that by tailoring the weight-update mechanism to the device’s hardware properties—especially by enforcing a strict LTD-only update on the G^−^ branch and scaling the initial step size—we can significantly enhance the network’s convergence speed, accuracy, and robustness against noise. This work not only provides an experimentally calibrated behavioral simulation platform for evaluating three-terminal synaptic arrays but also offers concrete design guidelines for hardware–algorithm co-optimization for future neuromorphic systems.

## 2. Research Methodology and Simulation Framework

The core of neuromorphic computing lies in leveraging the physical properties of synaptic devices to achieve efficient learning and inference, where the weight-update rules directly determine the network’s convergence behavior and final accuracy. Given the inherent nonlinearity and boundary constraints of conductance modulation in real oxide and GeO_x_-MXene synaptic transistors, establishing a parametric simulation framework that accurately captures these hardware characteristics is essential for algorithm–hardware co-design. This section elaborates on the mathematical foundation, update protocols, and optimization mechanisms of the framework, providing a rigorous methodological basis for the subsequent parameter sensitivity analysis.

### 2.1. Simulation Framework and Differential Weight Mapping Logic

In a crossbar array architecture based on synaptic devices, the conductance G of an individual device is physically constrained to a positive range [G_min_, G_max_]. This positivity restriction precludes the direct representation of negative synaptic weights using a conventional single-device mapping scheme, as required in standard neural network implementations. Inspired by the synergistic encoding of excitatory and inhibitory signals in biological synapses, the present framework adopts a differential mapping strategy using a pair of complementary synaptic devices, denoted as G^+^ and G^−^. The effective network weight W is then defined as the difference between the conductances of these two devices:

(1) $$W=G^{+}-G^{-}$$

This differential configuration enables the representation of both positive and negative weights through the linear combination of two positive conductances, thereby overcoming the inherent limitation of single-device mapping while preserving natural compatibility with bidirectional update operations. Weight increase can be achieved either by potentiating G^+^ (LTP) or by depressing G^−^ (LTD), and vice versa for weight decrease. This symmetry provides the architectural foundation for exploring a wide range of update polarity constraint strategies in the subsequent analysis.

The array size is adapted to match the weight scale of the Neural Circuit Policies (NCP) sparse network, where each weight node in the network corresponds to one pair of generic synaptic device units. The simulation does not solve the detailed device physics equations; instead, it abstracts the conductance update as an iterative mathematical process governed by the core parameters—update polarity, step-size coefficients, and nonlinearity exponents. This approach ensures computational efficiency while enabling a systematic scan of the hardware performance space. The validation task pipeline is designed following the benchmark standards for mainstream neuromorphic devices: basic functionality is first verified on prior benchmark tasks, and the optimized update protocols are then transferred to the ICRA 2020 LiDAR autonomous driving obstacle-avoidance dataset, where the NCP sparse network executes high-order spatiotemporal path recognition tasks to evaluate the generalization capability of the update rules in complex real-world scenarios. The NCP network is selected as the validation platform for three reasons. Its inherent sparsity maps naturally onto the limited resources of hardware arrays, its design for temporal decision-making aligns with the sequential pulse-based update scheme of synaptic devices, and its interpretable architecture facilitates tracing the impact of update rules on network outputs. While the validation is performed on NCP, the parametric update rules and optimization principles are general and transferable to other network topologies.

### 2.2. Exponential Update Mechanisms and Parameter Definitions for LTP/LTD

The evolution of synaptic device conductance under successive programming pulses typically exhibits a nonlinear saturating behavior, where the update step size gradually decays as the current conductance level approaches the boundaries. This phenomenon closely resembles the calcium-ion-dependent plasticity window observed in biological synapses. Based on the measured responses of oxide and GeO_x_-MXene-based synaptic transistors under hybrid optoelectronic stimulation, this work establishes the following generic exponential conductance-update fitting framework. For the positive synaptic device G^+^, the long-term potentiation (LTP) process increases the conductance following an exponential rise, with the update equation expressed as:

(2) $$G_{n+1}^{+}=G_{n}^{+}+A_{p}\cdot exp(-\beta_{p}\cdot \frac{G_{n}^{+}-G_{min}}{G_{max}-G_{min}})$$

Correspondingly, the long-term depression (LTD) process decreases the conductance following an exponential decay, with the update equation given by:

(3) $$G_{n+1}^{+}=G_{n}^{+}-A_{d}\cdot exp(-\beta_{d}\cdot \frac{G_{max}-G_{n}^{+}}{G_{max}-G_{min}})$$

where A_p_ and Ad are the first-step coefficients for LTP and LTD, respectively, determining the initial magnitude of the update amplitude; β_p_ and β_d_ are dimensionless nonlinearity exponents that modulate the decay rate of the step size as a function of the normalized conductance level. The normalization factor (G_n_^+^ − G_min_)/(G_max_ − G_min_) in the exponential term maps the conductance value onto the [0, 1] interval, ensuring comparability of the update dynamics across different device specifications. This exponential update function has been shown to accurately fit the measured LTP/LTD curves of various two-terminal and three-terminal synaptic devices, and is therefore widely adopted as a standard model for neuromorphic system-level simulations. The update logic for the negative synaptic device G^−^ is strictly symmetric to that of G^+^, requiring only the interchange of LTP and LTD operations and the inversion of the conductance variables. The overall network weight change is jointly determined by the positive and negative conductance changes:

(4) $$\Delta W=\Delta G^{+}\;-\;\Delta G$$

This expression is fully self-consistent with the differential mapping definition, ensuring mathematical closure of the weight-update procedure.

The baseline parameter set is aligned with the hardware measurement data from previous NCP task studies: G_max_ = 304.4 nS, G_min_ = 0.97 nS, A_p_ = 0.2107 nS, A_d_ = 1.1852 nS, β_p_ = 5.0480, and β_d_ = 1.8224. On this basis, the present work constructs a multi-dimensional parameter scanning system to systematically reveal the regulatory effects of each hardware parameter on network performance. Integer nonlinearity levels NL = 1–5 are introduced, corresponding to exponent coefficients β = 1–5, where β = 5 is closest to the hardware-measured value β_p_ = 5.0480, and β = 1 makes the exponential term approach a constant (i.e., near-linear update), with the intermediate levels uniformly interpolated to cover the continuous influence from weak to strong nonlinearity. Two physical cases for the sign of the LTD exponential term are distinguished (β > 0: natural decay of the update amount in the high-conductance region; β < 0: reverse amplification), providing a parametric comparison with the hardware-measured β_p_ = 5.0480. A first-step scaling factor λ ∈ {0.1,0.5,1,2,5} is additionally introduced, with values approximately uniformly distributed on a logarithmic axis to cover the complete behavioral spectrum from quasi-linear response (0.1×) to severe saturation (5×). This factor scales A_p_ and A_d_ proportionally to quantify the marginal effects of update amplitude on convergence speed and steady-state accuracy. Furthermore, G_max_ is scaled to 0.75× and 1.5× of its baseline, and G_min_ to 0.1× and 5×. Given that G_max_ and G_min_ differ by approximately two orders of magnitude, the former adopts relatively small deviations (±25% to +50%) to produce engineering-meaningful dynamic range changes, while the latter requires larger spans (×0.1 or ×5) to generate observable differences in the update formula—hence the chosen scaling factors. These boundary variations are used to examine the constraints imposed by the intrinsic device specifications on the update rules. The above parameter system provides a complete variable space for the layer-by-layer simulation analysis in Section 3.

### 2.3. Invalid Update Suppression Mechanism

The exponential update rules exhibit strong step-size asymmetry in the low- and high-conductance regions. When the network weight approaches the target value, a single update can easily cause overshoot, driving the weight away from the target and even inducing oscillation. Inspired by the weight-dependent modulation mechanism of spike-timing-dependent plasticity in biological synapses, the present framework introduces a posteriori invalid-update screening mechanism (denoted as isValid). This mechanism functions as a lightweight, adaptive gradient-clipping strategy. After each iteration, the absolute deviation between the current weight and the target is computed and compared before and after the update: if the deviation after the update is smaller than that before the update, the update is deemed valid and retained; otherwise, it is zeroed out. The decision logic is described by the following equations:

(5) $${\mathrm{diff}}_{1}=\left|\mathrm{target}-\left(G_{\mathrm{pre}}^{+}-G_{\mathrm{pre}}^{-}\right)\right|\;{\mathrm{diff}}_{2}=\left|\mathrm{target}-\left(G_{\mathrm{post}}^{+}-G_{\mathrm{post}}^{-}\right)\right|\;\mathrm{isValid}=\left\{\begin{array}{l} 1,{\;\mathrm{diff}}_{2}\text{<}{\mathrm{diff}}_{1}\;(\mathrm{update}\;\mathrm{retained})\\ 0,\;\mathrm{otherwise}\;(\mathrm{update}\;\mathrm{discarded},\;\mathrm{set}\;\mathrm{to}\;\mathrm{zero}) \end{array}\right.$$

This binary decision logic does not require modification of the mathematical form of the update rule itself; it performs only a local check at the end of each iteration, incurring extremely low computational overhead. By suppressing over-update events, the isValid mechanism significantly extends the effective learning window of the network within a limited number of iterations, particularly under strong nonlinearity or small step-size conditions, effectively improving the stability of weight convergence and the final accuracy. In the parameter optimization analyses that follow, the triggering rate of isValid and the retention rate of effective updates are used as key indicators to quantify the update efficiency under different parameter configurations.

## 3. Results and Discussion

An NCP simulation framework is established for the ICRA 2020 LiDAR autonomous driving obstacle-avoidance task, where network weights are constructed via differential conductance-pair mapping and the isValid mechanism is introduced to screen out ineffective updates that deviate from the target. Within this framework, the core tuning parameters of conductance updates directly dictate the convergence efficiency and the final inference accuracy of the network.

The analysis proceeds layer by layer, from single-parameter rules to device characteristics and then to array-level system effects. The investigation begins with a systematic scan of 15 conductance update polarity constraint strategies under a fixed baseline step size, aiming to identify the optimal update logic for the task. Based on the preferred strategies, a first-step scaling factor is then introduced to quantify the marginal impact of update amplitude on convergence speed, steady-state accuracy, and noise robustness. Moving beyond the ideal linear baseline model, the inherent exponential evolution characteristics of synaptic device conductance are exploited to unravel the potential perturbation mechanisms of nonlinearity on the aforementioned optimization outcomes, thereby providing a theoretical foundation for subsequent integration of experimental device data. On this basis, the regulatory effects of conductance boundaries—an intrinsic device specification—on the update rules are further examined to establish matching criteria between boundary parameters and update directions. Finally, the analysis is extended from single devices to multi-device arrays, where a preliminary simulation framework is constructed to inject device-to-device performance variability into the system evaluation.

### 3.1. Conductance Update Polarity Constraints on Weight Modulation

Based on the differential mapping framework established in Section 2.1 (W = G^+^ − G^−^), where LTP and LTD operations produce opposite effects on the weight depending on which channel they are applied to, this subsection systematically investigates the optimal polarity constraint strategies for weight updates.

In the existing differential-pair scheme, it remains unspecified whether a weight update should be applied to both devices in the pair or to only one of them. To address this, the present study defines a total of 15 conductance update strategies, among which 14 are polarity-constrained configurations (covering single-side freezing, single-constraint combination, dual unidirectional, and single-side unidirectional constraints) and the remaining one is the fully bidirectional strategy (which corresponds to the conventional update scheme without any polarity restriction) where both G^+^ and G^−^ are updated bidirectionally. The detailed configurations are grouped into the five categories listed in Table 1, and all baseline results are obtained under the default conductance boundaries.

**Figure 1:**
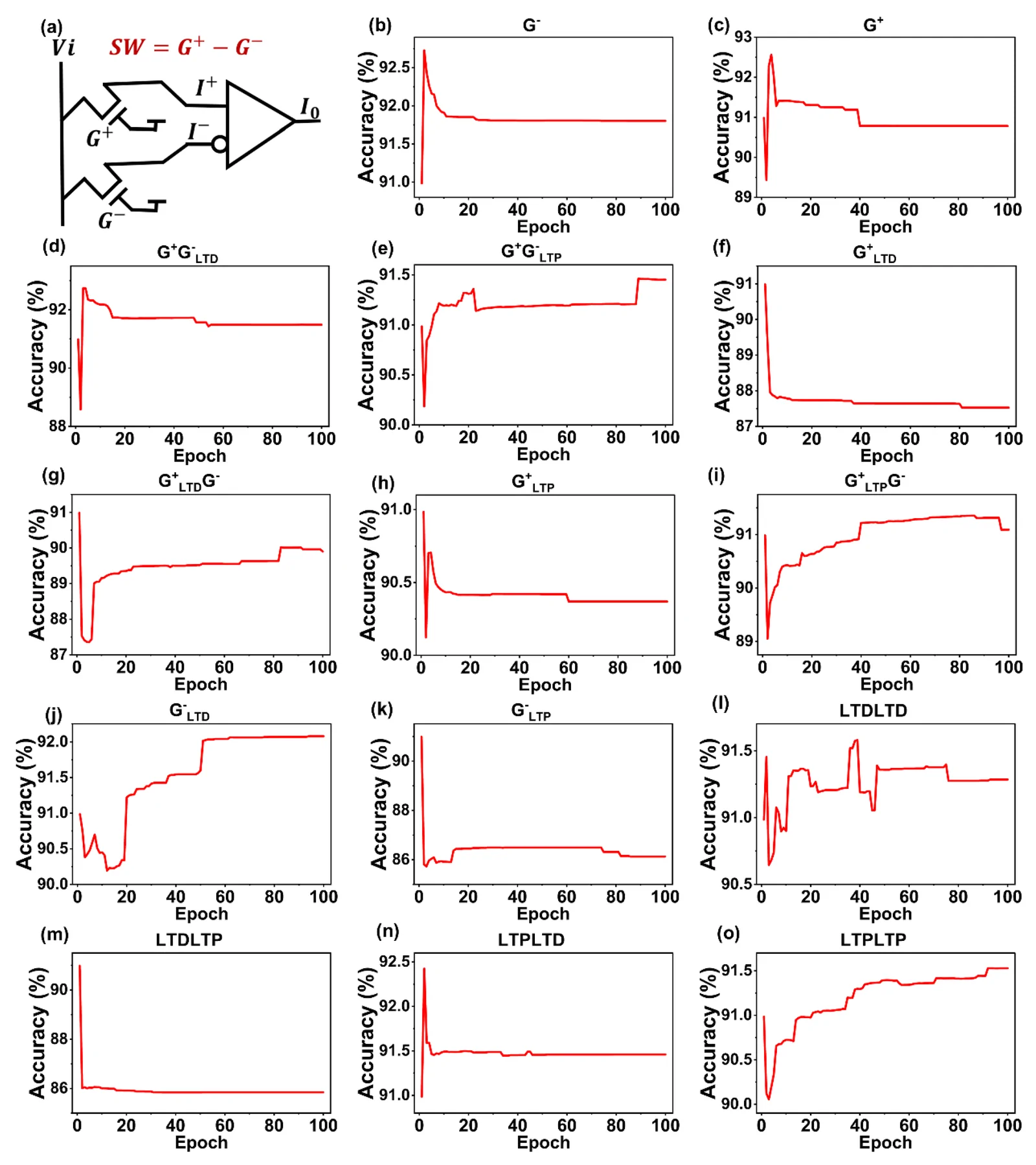
Training accuracy versus epoch for conductance update polarity constraint strategies under the differential weight mapping framework ($W=G^{+}-G^{-}$). (**a**) Schematic of the differential mapping architecture; (**b**) G^−^; (**c**) G^+^; (**d**) G^+^G^−^LTD; (**e**) G^+^G^−^LTP; (**f**) G^+^LTD; (**g**) G^+^LTDG^−^; (**h**) G^+^LTP; (**i**) G^+^LTPG^−^; (**j**) G^−^LTD; (**k**) G^−^LTP; (**l**) LTDLTD; (**m**) LTDLTP; (**n**) LTPLTD; (**o**) LTPLTP. Among all configurations, the G^−^LTD strategy (**j**) achieves the highest final accuracy (~92.08%), while LTDLTP (**m**) performs the worst (~85.85%), indicating that unidirectional LTD on with frozen yields optimal convergence.

The experimental results, as presented in Figure 1, reveal that the performance of the 15 strategies is fundamentally governed by whether the update direction aligns with the assigned role of the conductance matrix. Since G^−^ encodes the negative component of the weight, its decrement constitutes a critical degree of freedom for learning. Consequently, adopting LTD on the G^−^ side—which suppresses conductance and indirectly increases the weight—precisely matches this role. Conversely, applying LTP on G^−^ leads to monotonic weight decay and ultimately prevents network convergence.

Consistent with the above alignment principle, the single-side unidirectional G^−^LTD strategy—where G^+^ is frozen and G^−^ executes LTD only—ranks first with a final accuracy of 0.9208 and a peak effective-update count of 681. The advantage lies in the fact that LTD on G^−^ naturally amplifies the weight, while freezing G^+^ eliminates counteracting interference, forming an efficient “unidirectional precision modulation” mode. The G^+^G^−^LTD strategy follows closely with 0.9149, where normal updates on G^+^ provide additional positive modulation, achieving the best steady-state stability (fluctuation < 0.002) under bidirectional programming. In the single-side freezing group, the G^−^ (bidirectional) strategy (0.9175)—where G^+^ is frozen and G^−^ performs both LTP and LTD—further confirms the dominant role of the negative channel. In stark contrast, the LTD/LTP strategy yields the poorest performance (0.8585), because LTP on G^−^ further shrinks the negative weight component, leading to irreversible monotonic decay.

In summary, a clear conductance update guideline is established for the differential weight architecture: the G^−^ side must adopt LTD-only suppression updates, with LTP strictly prohibited; the G^+^ side may be frozen to minimize hardware overhead or perform normal bidirectional updates to enhance flexibility. The G^−^ LTD and G^+^G^−^ LTD strategies emerge as the preferred configurations, and all subsequent analyses on nonlinearity and step-size optimization are built upon these baselines. Confusion-matrix analysis further reveals that the recognition accuracy for small-angle left turns is slightly lower (~82%), which is directly attributable to the under-representation of this class in the training dataset.

### 3.2. Regulation of Update Stability by Scaling the Initial Step Size

To further investigate the regulatory effect of the conductance update amplitude, this study introduces a first-step scaling factor λ in {0.1, 0.5, 1, 2, 5}, with values approximately uniformly distributed on a logarithmic scale to cover the complete behavioral spectrum from quasi-linear response (0.1×) to severe saturation (5×). This factor proportionally scales the initial step sizes of both LTP and LTD, with the detailed parameter configurations listed in Table 2.

**Figure 2:**
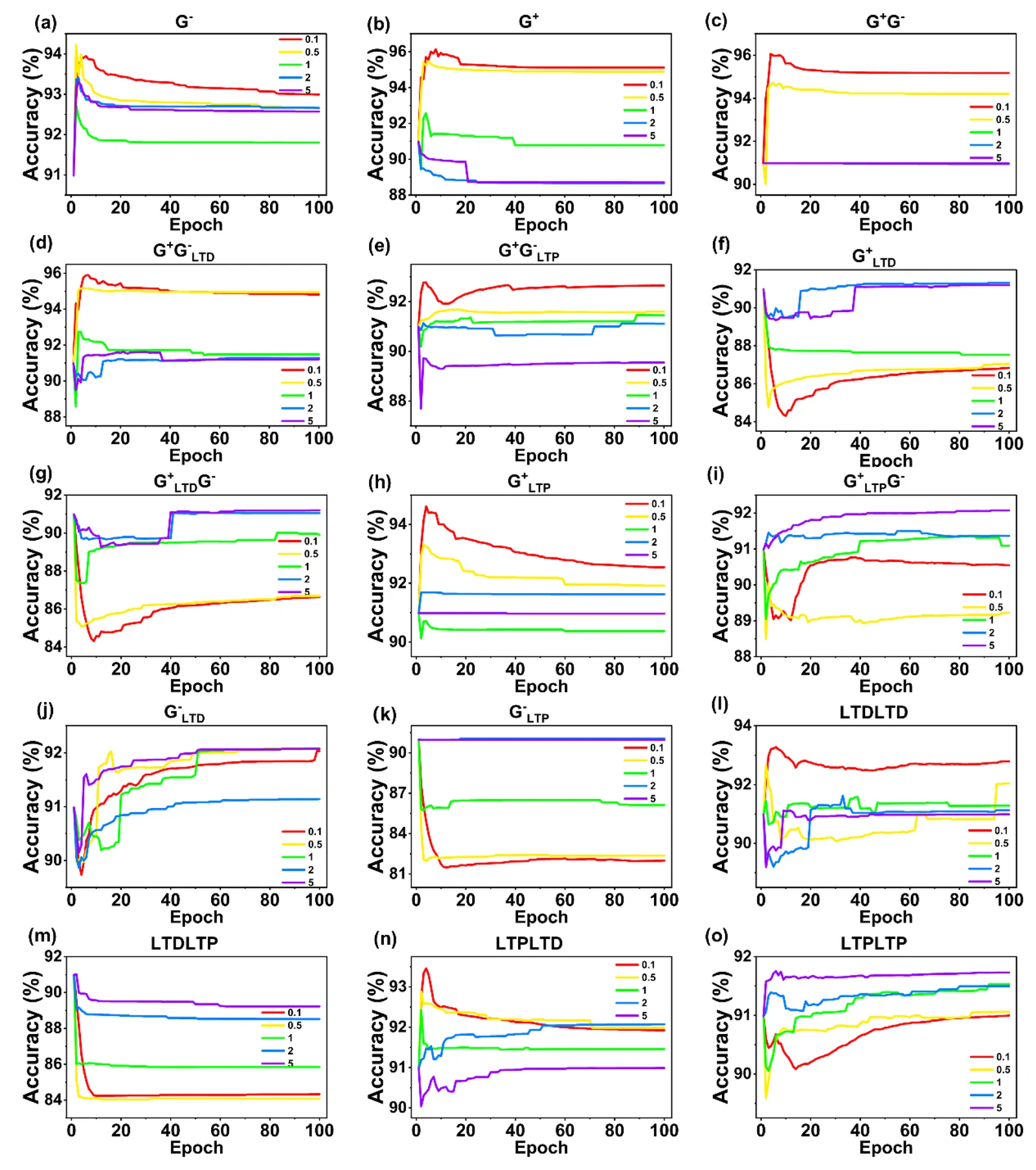
Under different first-step scaling factors λ ∈ {0.1, 0.5, 1, 2, 5}. (**a**) LTPLTP; (**b**) LTPLTD; (**c**) LTDLTP; (**d**) LTDLTD; (**e**) G^−^LTP; (**f**) G^−^LTD; (**g**) G^+^LTPG^−^; (**h**) G^+^LTP; (**i**) G^+^LTDG^−^; (**j**) G^+^LTD; (**k**) G^+^G^−^LTP; (**l**) G^+^G^−^LTD; (**m**) G^+^G^−^; (**n**) G^+^; (**o**) G^−^. The G^+^G^−^LTD strategy (**l**) achieves the highest final accuracy of 0.9481 under λ = 0.1, while the LTPLTP strategy (**a**) yields the lowest, confirming that unidirectional LTD on G^−^ combined with a small step size provides optimal convergence and noise robustness. (**c**) The blue and green lines overlap with the purple line and are therefore not visible separately.

As shown in Figure 2, the impact of step-size scaling on network performance exhibits a marked asymmetry. Among the 15 update strategies, 9 achieve their optimal results at λ = 0.1, indicating that a small step size effectively suppresses the nonlinear overshoot inherent in conductance updates of synaptic devices, bringing weight changes closer to the linear regime and reducing the probability of invalid updates being rejected by the isValid mechanism. Notably, the G^+^G^−^LTD strategy achieves a peak accuracy of 0.9590 at λ = 0.1, stabilizing at a final accuracy of 0.9481—the highest among all experiments. At λ = 0.5, this strategy still attains a final accuracy of 0.9481, with a fluctuation of less than 0.0003 over the last 50 iterations, demonstrating exceptional steady-state stability. In contrast, the fully bidirectional G^+^G^−^ strategy, where both matrices are updated simultaneously, suffers from an equivalent doubling of the effective update amplitude due to the superposition of the two step sizes. Consequently, most updates are flagged by the isValid mechanism as “deviating from the target,” effectively freezing the network weights; the accuracy remains at the baseline level of approximately 0.909 across all scaling factors. From the perspective of the conductance mechanism parameters, the first-step coefficient of LTP is Ap = 0.2107, while that of LTD is Ad = 1.1852—the latter being approximately 5.6 times larger than the former. This mechanistic asymmetry explains the high efficiency of LTD operations on the G^−^ channel: a smaller number of pulses is required to achieve an equivalent weight modulation, which meets the low-power requirements of neuromorphic arrays.

Collectively, these results reveal the core regulatory roles of update polarity and step-size parameters in array computational efficiency and robustness, and they also provide a baseline reference for the subsequent analysis of nonlinearity-induced perturbations to the update process.

### 3.3. Effect of Conductance Nonlinearity on Weight-Update Linearity

To further investigate the regulatory effect of conductance update nonlinearity, this study introduces a nonlinearity level parameter NL = 1–5, corresponding to exponent coefficients β = 1–5, as listed in Table 3. Two physical cases for the sign of the LTD exponential term are explicitly distinguished: LTD > 0 (positive exponent, natural decay of the update amount in the high-conductance region, which is purely hypothetical) and LTD < 0 (negative exponent, reverse amplification in the high-conductance region).

**Figure 3:**
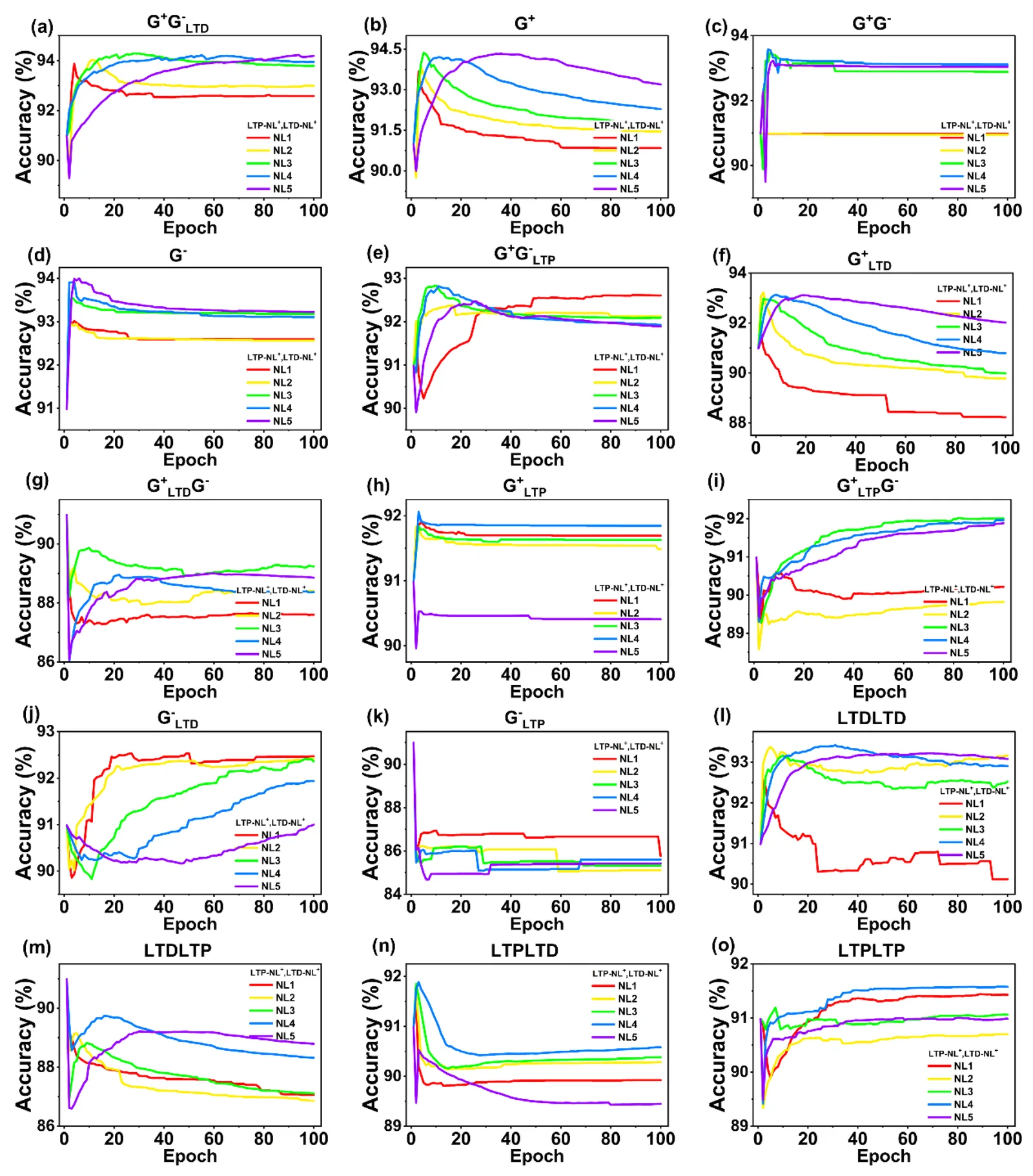
(**a**–**o**) Accuracy of 15 Strategies versus Iteration Count under $LTP-NL^{+},LTD-NL^{+}$. (**a**) G^−^; (**b**) G^+^; (**c**) G^+^G^−^; (**d**) G^+^G^−^LTD; (**e**) G^+^G^−^LTP; (**f**) G^+^LTD; (**g**) G^+^LTDG^−^; (**h**) G^+^LTP; (**i**) G^+^LTPG^−^; (**j**) G^−^LTD; (**k**) G^−^LTP; (**l**) LTDLTD; (**m**) LTDLTP; (**n**) LTPLTD; (**o**) LTPLTP. The G^+^G^−^LTD strategy (**d**) achieves the highest final accuracy, while the LTPLTP strategy (**o**) performs the worst, confirming that unidirectional LTD on G^−^ combined with a small step size provides optimal convergence and noise robustness. Under this condition, the G^+^G^−^LTD strategy (**d**) achieves the highest final accuracy, while the LTPLTP strategy (**o**) exhibits the poorest performance, indicating that unidirectional LTD constraints on G^−^combined with a moderate step size yield the best convergence.

**Figure 4:**
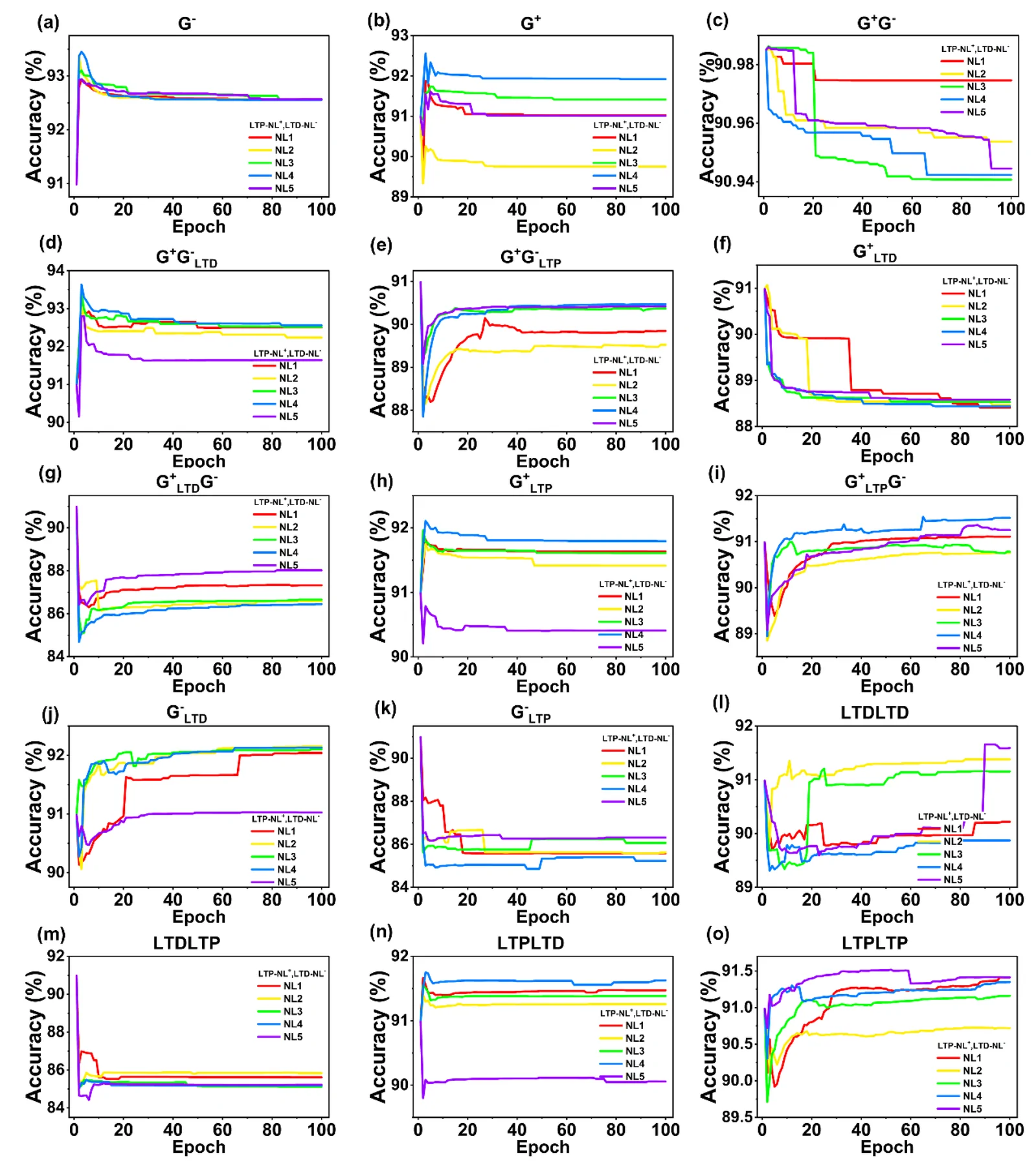
(**a**–**o**) Accuracy of 15 Strategies versus Iteration Count under $LTP-NL^{+},LTD-NL^{-}$ (**a**) G^−^; (**b**) G^+^; (**c**) G^+^G^−^; (**d**) G^+^G^−^LTD; (**e**) G^+^G^−^LTP; (**f**) G^+^LTD; (**g**) G^+^LTDG^−^; (**h**) G^+^LTP; (**i**) G^+^LTPG^−^; (**j**) G^−^LTD; (**k**) G^−^LTP; (**l**) LTDLTD; (**m**) LTDLTP; (**n**) LTPLTD; (**o**) LTPLTP. Interestingly, under the reversed noise level for LTD, the G^+^G^−^LTD strategy (**d**) still maintains superior performance, whereas the LTPLTP strategy (**o**) continues to perform the worst, suggesting that the advantage of unidirectional LTD on G^−^ is robust against variations in noise polarity.

As shown in Figure 3 and Figure 4, the impact of nonlinearity on network performance exhibits a pronounced sign dependence. Among the 15 update strategies, the G^+^G^−^LTD strategy under the LTD > 0 condition—where G^+^ performs normal bidirectional updates and G^−^ is restricted to LTD only—exhibits a unique nonlinear gain effect, with accuracy monotonically increasing from 0.9259 to 0.9419 as NL increases, reaching the highest value across all experiments. In contrast, the same strategy under the LTD < 0 condition drops to 0.9164 at NL = 5, showing an opposite degradation trend. Notably, the strategy that updates only the G^−^ matrix (normal linear update) exhibits the strongest steady-state robustness under the LTD < 0 condition, with accuracy stabilized at 0.9256 across all NLs and a variance below 0.0001, demonstrating complete immunity to the sign of nonlinearity. This result corroborates the unique advantage of asymmetric single-channel updates in the differential weight architecture: reducing redundant degrees of freedom effectively lowers the optimization difficulty and improves convergence quality within a limited number of iterations. However, when the update direction violates the matching principle—i.e., when LTP operations are applied to the G^−^ matrix (G^−^LTP strategy)—the accuracy plummets to approximately 0.854 regardless of the LTD sign, representing a drop of more than 6 percentage points from the initial accuracy of 0.9099. This confirms the catastrophic consequence of direction mismatch.

From the perspective of the conductance mechanism parameters, the exponential structure of LTD > 0 naturally shrinks the update amount in the high-conductance region (exp(−β ×(G_max_ − G)/(G_max_ − G_min_)) tends to be small), forming a conductance-dependent implicit regularization effect: when the network has already learned important weights (G^−^ in the high-conductance state), LTD updates are automatically suppressed to protect the acquired knowledge; when the weights are still in the secondary range (G^−^ close to G_min_), the update amount is fully released to accelerate convergence. The isValid statistics show that under LTD > 0, the G^+^G^−^LTD strategy has 618 updates rejected but achieves the highest accuracy (0.9419); under LTD < 0, zero updates are rejected but the performance drops to 0.9164. Furthermore, the validNum of the G^−^LTP strategy increases sharply with NL while the accuracy remains unchanged, further confirming the existence of spurious effective updates: individual weights may appear closer to the target, but the overall coordination of the network weights is disrupted.

The above results reveal the core regulatory role of the nonlinearity sign and its alignment with the update direction in array computational efficiency, and they also provide a clear algorithm-hardware co-design guideline for the device selection of neuromorphic hardware—prioritizing synaptic devices with LTD > 0.

### 3.4. Regulation of Update-Rule Performance by Conductance Boundaries (G_max_/G_min_)

With step size and nonlinearity fixed at their baseline values, this subsection further investigates the regulatory effects of the conductance boundary parameters G_max_ and G_min_ on the 15 polarity constraint strategies. G_max_ and G_min_ define the upper and lower limits of conductance modulation for the synaptic devices, respectively, and their difference (G_max_ − G_min_) directly determines the effective dynamic range of weight updates. In the experiments, G_max_ is adjusted to 0.75× and 1.5× of its baseline value, while G_min_ is adjusted to 0.1× and 5× of its baseline value. Only one parameter is varied at a time to isolate the independent contribution of each boundary parameter.

**Figure 5:**
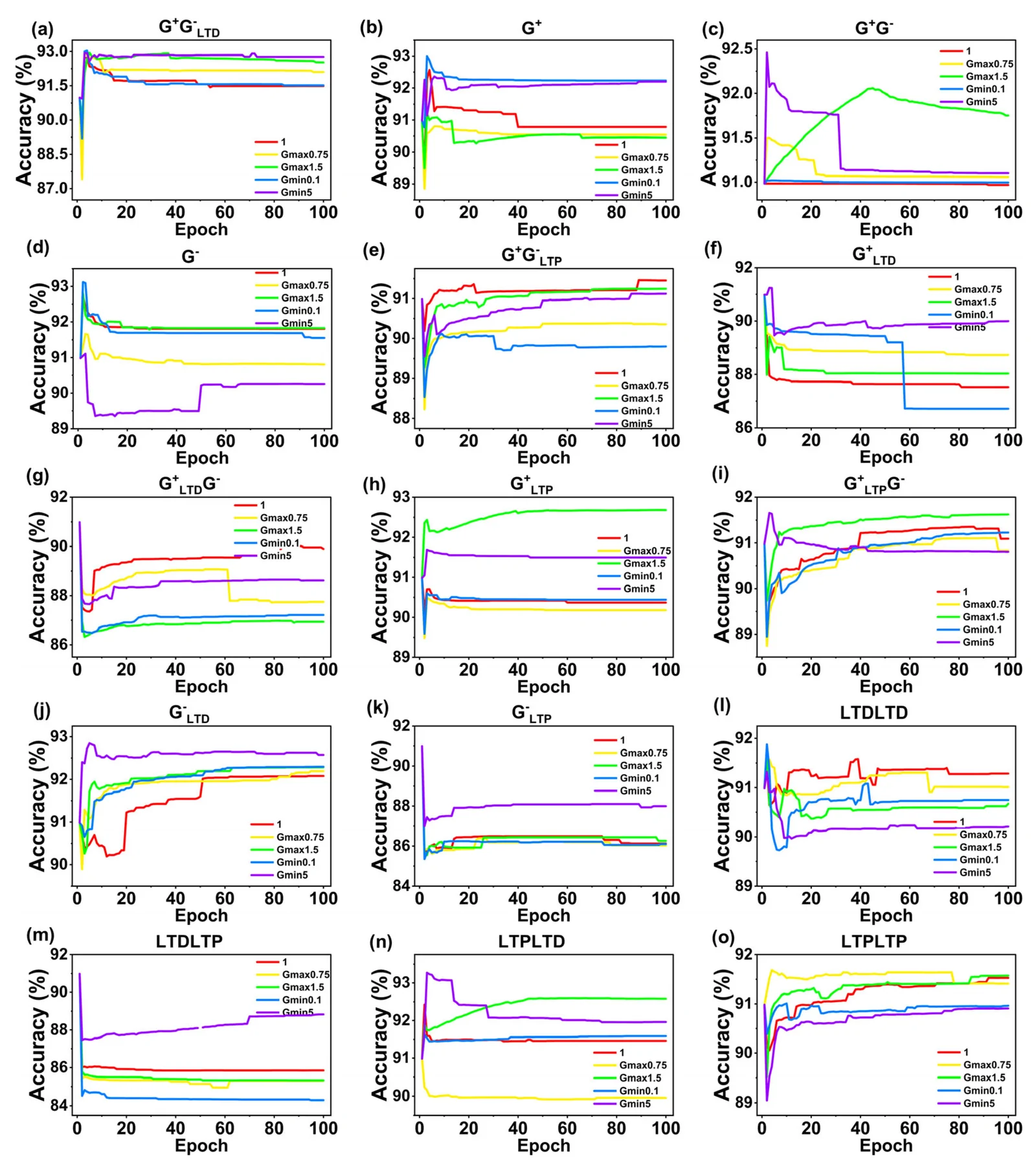
Accuracy of 15 strategies versus iteration count under different conductance boundary parameters (G_max_, G_min_). (**a**) G^−^; (**b**) G^+^; (**c**) G^+^G^−^; (**d**) G^+^G^−^LTD; (**e**) G^+^G^−^LTP; (**f**) G^+^LTD; (**g**) G^+^LTDG^−^; (**h**) G^+^LTP; (**i**) G^+^LTPG^−^; (**j**) G^−^LTD; (**k**) G^−^LTP; (**l**) LTDLTD; (**m**) LTDLTP; (**n**) LTPLTD; (**o**) LTPLTP. Under enlarged G_min_ (×5), the G^+^G^−^LTD strategy (**d**) achieves the highest final accuracy, while the LTPLTP strategy (**o**) performs the worst, confirming that raising the lower conductance bound effectively prevents over-attenuation in LTD-dominant updates and enhances convergence quality.

As shown in Figure 5, the optimization effect of the boundary parameters strictly depends on the directional property of the update rule. For LTP-dominant strategies, increasing G_max_ to 1.5× of the baseline significantly improves performance: the G^+^LTP strategy increases from a baseline accuracy of 0.9037 to 0.9268, and the LTP/LTD strategy improves from 0.9146 to 0.9258. The underlying mechanism is that a larger G_max_ provides more headroom for LTP operations. Conversely, for LTD-dominant strategies, an increase in G_max_ leads to a degradation in accuracy—for example, the LTD/LTD strategy drops from 0.9135 to 0.9067—because the absolute magnitude of the LTD step size is amplified, accelerating weight decay.

The variation in G_min_ has a more direct impact on LTD-dominant strategies. When G_min_ is raised to 5× of the baseline, the “floor” of LTD updates moves upward, effectively preventing excessive conductance attenuation. The G^+^G^−^LTD strategy improves from 0.9149 to 0.9277, reaching the highest accuracy across all boundary configurations; the G^−^LTD strategy also improves from 0.9208 to 0.9257, and maintains zero degradation or slight improvement under all parameter variations, demonstrating excellent passive fault tolerance. In contrast, reducing G_min_ to 0.1× allows LTD to reach lower conductance values, causing significant damage to LTD-dominant strategies—for instance, the LTD/LTP strategy drops from 0.8585 to 0.8429.

It is worth noting that boundary parameters cannot salvage structural defects caused by directional errors. For strategies such as G^−^LTP and LTD/LTP, regardless of how G_max_ or G_min_ is varied, the accuracy remains consistently in the range of 0.86–0.89, confirming that structural errors cannot be remedied by parameter tuning.

The results of this subsection indicate that the selection of conductance boundaries should follow a clear matching criterion: LTP-dominant updates favor a large G_max_ (1.5× of the baseline is recommended), while LTD-dominant updates favor a high G_min_ (5× of the baseline is recommended). Among all strategies, the G^−^LTD and G^+^G^−^LTD strategies exhibit the strongest robustness to boundary parameters, with accuracy fluctuations within ±0.005 under all parameter variations and, in most cases, no degradation or even improvement. This makes them particularly suitable for deployment scenarios where boundary parameters are subject to manufacturing deviations. This criterion provides direct guidance for the device specification design of neuromorphic hardware: if the algorithm layer adopts an LTD-dominant update mode, priority should be given to ensuring a sufficiently high G_min_, rather than blindly pursuing a large G_max_.

### 3.5. Preliminary Exploration of the Impact of Device-to-Device Performance Variations

Section 3.1, Section 3.2, Section 3.3 and Section 3.4 have systematically optimized parameters including conductance update polarity, step-size scaling, nonlinearity, and conductance boundaries at the single-device level. However, all the above analyses are based on the assumption of independent single-device simulation and have not yet verified the adaptability of the exponential LTP/LTD update rules to large-scale array parallel computing scenarios. This section examines the transferability of the single-device theory to array-level scenarios from two hierarchical perspectives: first, validating the effectiveness of the array-level G^+^/G^−^ separated update mechanism; second, evaluating the impact of different conductance physical parameters on the array-level training process through device-parameter ablation experiments.

The array-level simulation adopts the differential conductance mapping framework established in Section 2.1 and Section 2.2. The ICRA 2020 LiDAR autonomous driving obstacle-avoidance NCP sparse network is used as the array simulation carrier (input dimension: 64 × 7; NCP wiring: 12 interneurons + 8 command neurons + 1 motor neuron). All 23,715 weight parameters in the network are expanded into 47,430 independent memristor conductance pairs according to the differential mapping rule W = G^+^ − G^−^, where each conductance pair corresponds to one physical memristor device in the array. The array simulation maintains independent conductance state variables for each device. At each iteration, the update direction is calculated for every weight, and the corresponding device physical operation is executed accordingly. The conductance update strictly follows the exponential LTP/LTD formulas defined in Section 2.2, with the updated conductance values constrained within the physical boundaries [G_min_, G_max_]. The baseline device parameters are inherited from the measured LTP/LTD characteristic fitting results of the GeO_x_-MXene synaptic transistors.

Under the uniform array assumption, a complete update simulation is performed on the 23,715 weight elements (47,430 differential conductance elements). This step serves as a functional-correctness check: it verifies that the array-level software implementation is fully consistent with the single-device LTP/LTD update equations defined in Section 2.2, and that no programming logic errors, weight mapping errors, or boundary clipping errors exist. The results show 23,715 valid updates and 0 invalid updates, confirming the correctness of the implementation rather than serving as evidence of scalability. The scalability and adaptability arguments are instead supported by the subsequent ablation experiments presented below.

Having confirmed the validity of the array-level update mechanism, this subsection addresses the process variation issues in actual chip manufacturing by conducting conductance parameter ablation experiments to evaluate the impact of G_max_ and G_min_ deviations from their baseline values on the array-level training process. With the baseline parameters as reference, different scaling perturbations are applied to G_max_ and G_min_, and the Jaccard similarity index is recorded at each of the complete 50 epochs of array-level online training. Since some extreme scaling ratios lead to complete training divergence (the 0.1×, 0.3×, 0.9×, 2.0×, and 5.0× groups for G_max_, and the 0.1×, 0.9×, 1.5×, and 5.0× groups for G_min_ exhibit abnormal Jaccard metrics with no steady-state trend), only the groups that achieve stable convergence are included in the comparative analysis.

**Figure 6:**
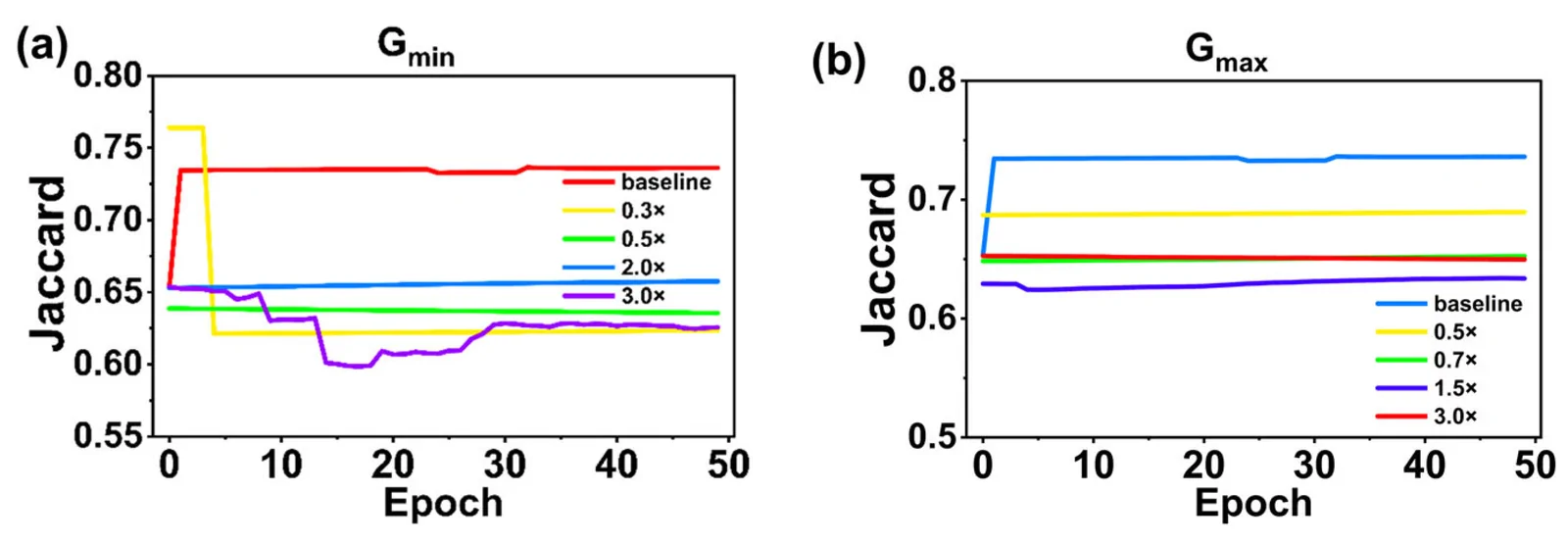
(**a**) Variation of the Jaccard index with training epochs under different scaling factors of G_max_. The NCP network (23,715 weights mapped to 47,430 differential conductance pairs) was trained with LTP/LTD device updates on the ICRA 2020 LiDAR collision-avoidance dataset. Only groups achieving stable convergence (0.5×, 0.7×, 1.5×, 3.0×) are plotted; other scaling ratios led to training divergence. The red curve represents the baseline configuration with unmodified G_max_ = 304.4 nS. (**b**) Variation of the Jaccard index with training epochs under different scaling factors of G_min_. Groups with divergent training are discarded; only convergent configurations (0.3×, 0.5×, 2.0×, 3.0×) are retained. The red curve represents the baseline with unmodified G_min_ = 0.97 nS. In contrast to the smooth degradation observed under G_max_ scaling, extreme G_min_ deviations induce abrupt performance collapse and mid-training instability.

Figure 6a shows the variation in the Jaccard index with training epochs under different G_max_ scaling ratios. All curves rise rapidly to the steady-state range in the early training stage (Epochs 0–5), without severe oscillations, indicating that the model quickly converges. After entering the steady-state stage (Epochs 5–50), the five curves exhibit stable, non-overlapping hierarchical ordering: the baseline group (red curve) achieves the highest steady-state Jaccard index, stably maintained in the range of 0.73–0.74, representing the optimal performance; the 0.5× group (orange) ranks second, with a steady-state value of approximately 0.69–0.71; the 0.7× group (yellow) and the 3.0× group (blue) exhibit similar performance, with steady-state Jaccard indices around 0.65–0.67; the 1.5× group (green) shows the lowest steady-state performance, stabilizing in the range of 0.63–0.65. Throughout the entire training period, all curves remain smooth and stable, with no sudden performance drops or significant oscillations, indicating that deviations in G_max_ only cause uniform and gradual accuracy degradation while the training process remains overall stable.

## 4. Conclusions

In this work, we have established an experimentally calibrated behavioral simulation framework for neuromorphic arrays based on differential conductance-pair mapping of synaptic devices, integrating exponential LTP/LTD update rules with an a posteriori isValid screening mechanism to systematically investigate how update polarity, step size, nonlinearity, and conductance boundaries regulate network computational efficiency. The study defines a clear matching criterion for conductance update direction in the differential weight architecture—the G^−^ side must strictly adopt unidirectional LTD inhibition updates while the G^+^ side can either be frozen or perform bidirectional updates—under which the proposed G^−^LTD and G^+^G^−^LTD strategies achieve high accuracies of 0.9208 and 0.9481, respectively, along with excellent noise robustness. Further analysis reveals that under the LTD > 0 condition, the G^+^G^−^LTD strategy exhibits a unique nonlinear gain effect, with accuracy monotonically increasing with nonlinearity level up to 0.9419, providing direct guidance for prioritizing synaptic devices with LTD > 0 in hardware selection. The study also establishes coupled design criteria between device specifications and update rules—LTP-dominant updates benefit from a large G_max_ while LTD-dominant updates benefit from a high G_min_—and the G^−^LTD and G^+^G^−^LTD strategies show accuracy fluctuations within ±0.005 across the tested boundary variations, suggesting that LTD-dominant modes should prioritize a sufficiently high G_min_ over a large G_max_. All optimization mechanisms are fully validated on the ICRA 2020 LiDAR autonomous driving obstacle-avoidance task, providing clear algorithm–hardware co-design guidelines for future neuromorphic systems: prioritize synaptic devices with LTD > 0, ensure a moderately elevated G_min_, and allocate processing resources asymmetrically toward the LTD side to achieve an optimal balance between manufacturing cost and array computational performance.

## Figures and Tables

**Table 1:** Conductance Update Constraint Strategies.

Category	Strategy	G^+^ Mode	G^−^ Mode	Response for W ↑	Response for W ↓
Single-side freezing	G^+^	LTP + LTD	Frozen	G^+^LTP	G^+^LTD
	G^−^	Frozen	LTP + LTD	G^−^LTD	G^−^LTP
Single-constraint combination	G^+^G^−^LTD	LTP + LTD	LTD only	G^+^LTP + G^−^LTD	G^+^LTD
	G^+^G^−^LTP	LTP + LTD	LTP only	G^+^LTP	G^+^LTD + G^−^LTP
	G^+^LTPG^−^	LTP only	LTP + LTD	G^+^LTP + G^−^LTD	G^−^LTP
	G^+^LTDG^−^	LTD only	LTP + LTD	G^−^LTD	G^+^LTD + G^−^LTP
Dual unidirectional	G^+^LTDG^−^LTP	LTD only	LTP only	Not available	G^+^LTD + G^−^LTP
	G^+^LTPG^−^LTD	LTP only	LTD only	G^+^LTP + G^−^LTD	Not available
	G^+^LTDG^−^LTD	LTD only	LTD only	G^−^LTD	G^+^LTD
	G^+^LTPG^−^LTP	LTP only	LTP only	G^+^LTP	G^−^LTP
Single-side unidirectional	G^+^LTP	LTP only	Frozen	G^+^LTP	Not available
	G^+^LTD	LTD only	Frozen	Not available	G^+^LTD
	G^−^LTP	Frozen	LTP only	Not available	G^−^LTP
	G^−^LTD	Frozen	LTD only	G^−^LTD	Not available
Full bidirectional	G^+^G^−^	LTP + LTD	LTP + LTD	G^+^LTP + G^−^LTD	G^+^LTD + G^−^LTP

Note: ↑ and ↓ denote an increase and a decrease, respectively.

**Table 2:** Parameter Table for Proportionally Scaled Initial Step Lengths.

Scaling Factor λ	LTP Step Coefficient $A_{p}$	LTD Step Coefficient $A_{d}$	Description
0.1×	0.02107	0.11852	Proportionally reduced; smoothest updates
0.5×	0.10535	0.59261	Proportionally reduced
1× (baseline)	0.21070	1.18522	Baseline; default step size
2×	0.42140	2.37044	Proportionally amplified
5×	1.05350	5.92610	Proportionally amplified; most aggressive updates

**Table 3:** Parameter Settings for Different Nonlinearity Levels.

Nonlinearity Level (NL)	LTP Exponent $\beta_{p}$	LTD > 0: $\beta_{d}$	LTD < 0: $\beta_{d}$
1	1	+1	−1
2	2	+2	−2
3	3	+3	−3
4	4	+4	−4
5	5	+5	−5

## Data Availability

The data presented in this study are available on request from the corresponding author.

## References

[1] Backus J. (1978). Can Programming Be Liberated from the von Neumann Style?. Commun. ACM.

[2] Horowitz M. Computing’s Energy Problem (and What We Can Do about It). Proceedings of the IEEE International Solid-State Circuits Conference (ISSCC).

[3] Salahuddin S., Ni K., Datta S. (2018). The Era of Hyper-Scaling in Electronics. Nat. Electron..

[4] Jeong D.S., Kim K.M., Kim S., Choi B.J., Hwang C.S. (2016). Memristors for Energy-Efficient New Computing Paradigms. Adv. Electron. Mater..

[5] LeCun Y., Bengio Y., Hinton G. (2015). Deep Learning. Nature.

[6] Indiveri G., Liu S.-C. (2015). Memory and Information Processing in Neuromorphic Systems. Proc. IEEE.

[7] Schuman C.D., Potok T.E., Patton R.M., Birdwell J.D., Dean M.E., Rose G.S., Plank J.S. (2017). A Survey of Neuromorphic Computing and Neural Networks in Hardware. arXiv.

[8] Sebastian A., Le Gallo M., Khaddam-Aljameh R., Eleftheriou E. (2020). Memory Devices and Applications for In-Memory Computing. Nat. Nanotechnol..

[9] Yang J.J., Strukov D.B., Stewart D.R. (2013). Memristive Devices for Computing. Nat. Nanotechnol..

[10] Ielmini D., Wong H.-S.P. (2018). In-Memory Computing with Resistive Switching Devices. Nat. Electron..

[11] Kim S., Lee Y., Kim H.-D., Choi S.-J. (2020). Parallel Weight Update Protocol for a Carbon Nanotube Synaptic Transistor Array for Accelerating Neuromorphic Computing. Nanoscale.

[12] Krauhausen I., Coen C.T., Spolaor S., Gkoupidenis P., van de Burgt Y. (2023). Brain-Inspired Organic Electronics: Merging Neuromorphic Computing and Bioelectronics Using Conductive Polymers. Adv. Funct. Mater..

[13] Liu Y., Yang W., Yan Y., Wu X., Wang X., Zhou Y., Hu Y., Chen H., Guo T. (2020). Self-Powered High-Sensitivity Sensory Memory Actuated by Triboelectric Sensory Receptor for Real-Time Neuromorphic Computing. Nano Energy.

[14] Hasler P., Diorio C., Minch B.A., Mead C., Tesauro G., Touretzky D.S., Leen T.K. (1995). Single Transistor Learning Synapses. Advances in Neural Information Processing Systems 7.

[15] Cao Y., Zhao T., Liu C., Zhao C., Gao H., Huang S., Li X., Wang C., Liu Y., Lim E.G. (2023). Neuromorphic Visual Artificial Synapse In-Memory Computing Systems Based on GeOx-Coated MXene Nanosheets. Nano Energy.

[16] Hu L., Yang J., Wang J., Cheng P., Chua L.O., Zhuge F. (2021). All-Optically Controlled Memristor for Optoelectronic Neuromorphic Computing. Adv. Funct. Mater..

[17] Hu L., Shao J., Wang J., Cheng P., Zhang L., Chai Y., Ye Z., Zhuge F. (2024). In Situ Cryptography in a Neuromorphic Vision Sensor Based on Light-Driven Memristors. Appl. Phys. Rev..

[18] Cao Y., Zhao C., Zhao T., Sun Y., Liu Z., Li X., Yin L., Gu J., Ren H., Geng X. (2023). Brain-Like Optoelectronic Artificial Synapse with Ultralow Energy Consumption Based on MXene Floating-Gate for Emotion Recognition. J. Mater. Chem. C.

[19] Xia Q., Yang J.J. (2019). Memristive Crossbar Arrays for Brain-Inspired Computing. Nat. Mater..

[20] Zidan M.A., Strachan J.P., Lu W.D. (2018). The Future of Electronics Based on Memristive Systems. Nat. Electron..

[21] Yu R., Li E., Wu X., Yan Y., He W., He L., Chen J., Chen H., Guo T. (2020). Electret-Based Organic Synaptic Transistor for Neuromorphic Computing. ACS Appl. Mater. Interfaces.

[22] Wang P., Li J., Xue W., Ci W., Jiang F., Shi L., Zhou F., Zhou P., Xu X. (2024). Integrated In-Memory Sensor and Computing of Artificial Vision Based on Full-vDW Optoelectronic Ferroelectric Field-Effect Transistor. Adv. Sci..

[23] Wang J., Yang B., Dai S., Guo P., Gao Y., Li L., Guo Z., Zhang J., Zhang J., Huang J. (2023). Weak Light-Stimulated Synaptic Transistors Based on MoS_2_/Organic Semiconductor Heterojunction for Neuromorphic Computing. Adv. Mater. Technol..

[24] Dang B., Liu K., Wu X., Yang Z., Xu L., Yang Y., Huang R. (2023). One-Phototransistor-One-Memristor Array with High-Linearity Light-Tunable Weight for Optic Neuromorphic Computing. Adv. Mater..

[25] Lechner M., Hasani R., Amini A., Henzinger T.A., Rus D., Grosu R. (2020). Neural Circuit Policies Enabling Auditable Autonomy. Nat. Mach. Intell..

